# General anesthesia *versus* conscious sedation in mechanical thrombectomy for patients with acute ischemic stroke: systematic review and meta-analysis

**DOI:** 10.1055/s-0044-1785693

**Published:** 2024-04-12

**Authors:** Ana Clara Felix De Farias Santos, Luciano Lobão Salim Coelho, Guilherme de Carvalho Caldas, Luziany Carvalho Araújo, Vivian Dias Baptista Gagliardi, Leonardo Augusto Carbonera

**Affiliations:** 1 Universidad Privada Franz Tamayo, Facultad de Ciencias de la Salud, La Paz, Bolivia.; 2 UDI Hospital Rede D'Or São Luiz, São Luís MA, Brazil.; 3 Neurosurgical Innovations and Training Center, WCMC, New York, New York, United States.; 4 Ebserh, Universidade Federal de Pernambuco, Hospital das Clínicas,, Recife PE, Brazil.; 5 Santa Casa de Misericórdia de São Paulo, São Paulo SP, Brazil.; 6 Hospital Moinhos de Vento, Serviço de Neurologia e Neurocirurgia, Porto Alegre RS, Brazil.

**Keywords:** Anesthesia, General, Conscious Sedation, Ischemic Stroke, Thrombectomy, Anestesia Geral, Sedação Consciente, AVC Isquêmico, Trombectomia

## Abstract

**Background** After recently published randomized clinical trials, the choice of the best anesthetic procedure for mechanical thrombectomy (MT) in acute ischemic stroke (AIS) due to large vessel occlusion (LVO) is not definite.

**Objective** To compare the efficacy and safety of general anesthesia (GA) *versus* conscious sedation (CS) in patients with AIS who underwent MT, explicitly focusing on procedural and clinical outcomes and the incidence of adverse events.

**Methods** PubMed, Embase, and Cochrane were systematically searched for randomized controlled trials (RCTs) comparing GA *versus* CS in patients who underwent MT due to LVO-AIS. Odds ratios (ORs) were calculated for binary outcomes, with 95% confidence intervals (CIs). Random effects models were used for all outcomes. Heterogeneity was assessed with I2 statistics.

**Results** Eight RCTs (1,300 patients) were included, of whom 650 (50%) underwent GA. Recanalization success was significantly higher in the GA group (OR 1.68; 95% CI 1.26–2.24; *p* < 0.04) than in CS. No significant difference between groups were found for good functional recovery (OR 1.13; IC 95% 0.76–1.67; *p* = 0.56), incidence of pneumonia (OR 1.23; IC 95% 0.56- 2,69; *p* = 0.61), three-month mortality (OR 0.99; IC 95% 0.73–1.34; *p* = 0.95), or cerebral hemorrhage (OR 0.97; IC 95% 0.68–1.38; *p* = 0.88).

**Conclusion** Despite the increase in recanalization success rates in the GA group, GA and CS show similar rates of good functional recovery, three-month mortality, incidence of pneumonia, and cerebral hemorrhage in patients undergoing MT.

## Resumo

**Antecedentes** A trombectomia mecânica (TM) é o padrão de tratamento para pacientes com acidente vascular cerebral isquêmico agudo (AVCI) devido à oclusão de grandes vasos (OGV). No entanto, ainda não está claro qual é o procedimento anestésico mais benéfico para a TM.

**Objetivo** Nosso objetivo foi comparar a eficácia e a segurança da anestesia geral (AG) *versus* sedação consciente (SC) em pacientes com AVCI submetidos à TM, focando especificamente nos resultados procedimentais e clínicos, bem como na incidência de eventos adversos.

**Métodos** Foram realizadas buscas sistemáticas nas bases PubMed, Embase e Cochrane por ensaios clínicos randomizados (ECRs) comparando AG *versus* SC em pacientes submetidos à TM devido a AVCI por OGV. Razões de chances (ORs) foram calculadas para desfechos binários, com intervalos de confiança de 95% (ICs). Modelos de efeitos aleatórios foram usados para todos os resultados. A heterogeneidade foi avaliada com estatísticas I2.

**Resultados** Oito ensaios clínicos randomizados (1.300 pacientes) foram incluídos, dos quais 650 (50%) foram submetidos à AG. O sucesso da recanalização foi significativamente maior no grupo AG (OR 1,68; IC 95% 1,26–2,24; *p* < 0,04) em comparação com SC. No entanto, não houve diferença significativa entre os grupos para recuperação funcional adequada (OR 1,13; IC 95% 0,76–1,67; *p* = 0,56), incidência de pneumonia (OR 1,23; IC 95% 0,56- 2,69; *p* = 0,61), mortalidade em três meses (OR 0,99; IC 95% 0,73- 1,34; *p* = 0,95) ou hemorragia cerebral (OR 0,97; IC 95% 0,68- 1,38; *p* = 0,88).

**Conclusão** Apesar do aumento significativo nas taxas de sucesso de recanalização no grupo AG, AG e SC mostram taxas semelhantes de recuperação funcional, mortalidade, pneumonia e hemorragia em pacientes com AVCI submetidos à TM.

## INTRODUCTION

Mechanical thrombectomy is indicated for patients with acute ischemic stroke due to a large artery occlusion in the anterior or posterior circulation who can be treated within 24 hours of the time last known to be well, whether or not they receive IV tPA for the same ischemic stroke event. 1

Mechanical thrombectomy can be performed with general anesthesia (GA) or conscious sedation (CS). The choice between anesthesia management is usually individualized based on patient and procedural factors and resource availability, 2 highlighting the selection's uncertainty. GA offers a still patient and a secure airway, the ability to institute controlled apnea, and the ability to fully control procedural pain. In contrast, CS has a shorter time for treatment initiation and allows neurologic examination during and after the procedure. The best evidence suggests that GA with optimal hemodynamic control may improve technical success 3 and functional outcomes. 4

The last meta-analysis included seven randomized trials of patients who underwent mechanical thrombectomy for anterior circulation ischemic stroke with GA versus non-GA techniques (local anesthesia, CS). 4 GA improved the recanalization rate and increased the functional independence rate (modified Rankin Scale 0 to 2) at three months.

However, these findings come from observational and preliminary studies 5 6 and several randomized trials that reported either no difference or a slight improvement in infarct size or other clinical outcomes with GA, while some retrospective and prospective observational studies have reported worse outcomes with GA for mechanical thrombectomy. A recent 2023 randomized clinical trial explored the impact of anesthesia or sedation on periprocedural complications and functional outcomes. 7 This study revealed that neither anesthesia nor procedural sedation significantly influenced these outcomes. The best anesthetic strategy during mechanical thrombectomy is still debatable in this context.

Therefore, this study presents a systematic review and meta-analysis comparing the effectiveness and safety of GA versus CS in patients who underwent a mechanical thrombectomy.

## METHODS

The Systematic review and meta-analysis were performed following the Cochrane Collaboration's tool for assessing the risk of bias and the Preferred Reporting Items for Systematic Reviews and Meta-Analysis (PRISMA) statement. 8 The study protocol was registered in the International Prospective Register of Systematic Reviews (PROSPERO) with the registration number CRD42023439944.

### Eligibility criteria and data extraction

Studies with the following eligibility criteria were included for analysis:

- randomized controlled trials (RCTs);
- comparing GA with CS;
- enrolling patients with AIS; and
- reporting at least one of the outcomes of interest.

Non-randomized studies and trials without a control group were excluded; studies with overlapping populations with the most significant number of patients were included.

Two authors (A.C.F.F.S and L.L.S.C.) independently extracted data following prespecified criteria for search, data extraction, and quality assessment methods. Disagreements were resolved by consensus between the two authors and the senior author (A.C.F.F.S., L.L.S.C. and L.A.C.).

### Search strategy

PubMed, Embase, and Cochrane Library were systematically searched on June 22, 2023, for studies published solely in English. The search strategy included the following terms: 'acute ischemic stroke,' 'ischemic stroke,' 'posterior circulation stroke,' 'endovascular therapy,' 'endovascular treatment,' 'thrombectomy,' 'vertebrobasilar stroke,' 'general anesthesia,' 'general anesthesia,' 'Non-general anesthesia,' 'conscious sedation,' 'moderate sedation, ''local anesthesia,' 'anesthesia care.' Additionally, the reference lists of all included studies and meta-analyses were manually assessed for additional studies. The search strategy is detailed in Supplementary Material ( https://www.arquivosdeneuropsiquiatria.org/wp-content/uploads/2024/04/ANP-2023.0290-Supplementary-Material-atualizado.docx ), Supplementary Material Table S1 .

### Endpoints

Outcomes of interest were:

- recanalization success;
- good functional recovery;
- 3- month mortality;
- cerebral hemorrhage; and
- pneumonia.

### Quality assessment

The Cochrane Collaboration's tool for risk of bias in randomized trials (ROB 2) was used to assess the quality assessment of individual studies. 9 Two authors (A.C.F.F.S. and V.D.B.C) conducted the quality assessment independently. Each trial was rated as having a high, low, or unclear risk of bias in the five domains: randomization process, deviations from the intended interventions, missing outcomes, measurement of the outcome, and selection of reported results. The quality of evidence was analyzed according to the Grading of Recommendations, Assessment, Development, and Evaluation (GRADE). 10 The outcomes were labeled with very low, low, moderate, or high-quality evidence based on five domains: risk of bias, inconsistency of results, imprecision, publication bias, and magnitude of treatment effects. Funnel plots of individual study weights versus point estimates were used to detect evidence of publication bias.

### Sensitivity analysis and meta-regression

We performed a leave-one-out sensitivity analysis for recanalization success, good functional recovery, three-month mortality, and cerebral hemorrhage. We removed each study from the outcome assessment to determine whether the results depended on a single study. Furthermore, we conducted a meta-regression to investigate any correlation between the National Institute of Health Stroke Scale (NIHSS) score and recanalization success.

### Statistical analyses

Odds ratio (OR) with 95% confidence intervals (CI) was computed to compare the incidence of treatment effect for binary endpoints. Cochran Q test and I2 statistics were used to analyze heterogeneity; p-values were considered low heterogeneity if *p* >10 and I2 <25%. DerSimonian and Laird random-effects models were used.

Review Manager 5.4 (Nordic Cochrane Centre, The Cochrane Collaboration, Copenhagen, Denmark) and R statistical software, version 4.3.0 (R Foundation for Statistical Computing), were used to perform the statistical analysis.

## RESULTS

### Study selection and characteristics

**Figure 1:**
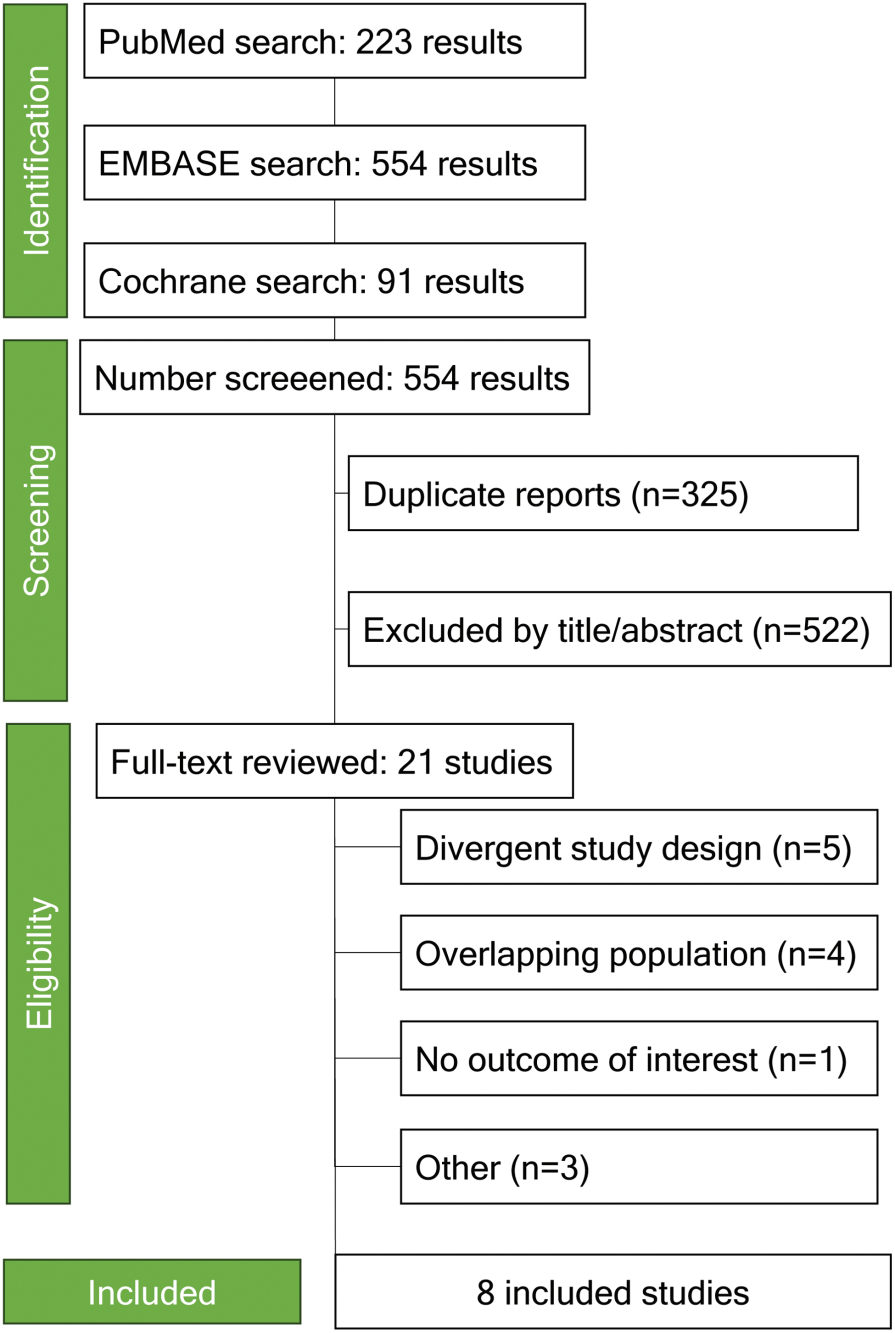
Preferred Reporting Items for Systematic Reviews and Meta-Analysis (PRISMA) flow diagram of study screening and selection.

As detailed in Figure 1 , 868 studies were found. After removing duplicates and abstract screening, 21 studies were fully assessed for inclusion. A total of 8 studies and 1,300 patients were included, of whom 650 (50%) underwent GA. 3 7 11 12 13 14 15 16 Additional population characteristics are reported in Table 1 .

**Table 1:** Baseline characteristics of included studies

Study	*n*	Follow-up	Population	Age, years (SD)	Female, n (%)	ASPECTS, median	NIHSS score, median (IQR)	mRS 0, n (%)	M1, n (%)	Hypertension n, (%)
Chabanne 2023 7	273	90 days	Large vessel, AC	GA: 72.0 (13.2) CS: 71.3 (14.4)	GA: 70 (51.9) CS: 72 (52.2)	GA: 8 (7–9) CS: 8 (7–9)	GA: 16 (11–20) CS: 15 (11–20)	NA	GA: 86 (63.7) CS: 84 (60.9)	NA
Hendén 2017 14	90	90 days	Large vessel, AC	GA: 73 (65–80) CS: 72 (66–82)	GA: 19 (42) CS: 22 (49)	GA: 10 (8–10) CS: 10 (9–10)	GA: 20 (15.5–23) CS: 17 (14–20.5)	NA	GA: 26 (58) CS: 26 (58)	GA: 27 (60) CS: 22 (49)
Hu 2021 16	139	90 days	Vertebro basilar stroke	GA: 72.1 (6.8) CS: 71.9 (7.5)	GA: 34 (47.2) CS: 33 (49.25)	NA	NA	GA: 55 (76.39) CS: 50 (74.63)	NA	GA: 34 (47.22) CS: 31 (46.27)
Liang 2023 11	87	90 days	Vertebro basilar stroke	GA: 64 (11) CS: 60 (13)	GA: 10 (23.3) CS: 6 (13.6)	NA	GA: 16 (12–21) CS: 15 (12–18)	NA	NA	GA: 32 (74.4) CS: 31 (70.5)
Maurice 2022 3	345	6 months	Large vessel, AC	GA: 70.8 (+13.0) CS: 72.6 (+12.3)	GA: 80 (47) CS: 77 (44)	NA	GA: 16 ± 6 CS: 16 ± 5	NA	GA: 99 (59) CS: 109 (62)	GA: 97 (57) CS: 124 (70)
Ren 2020 12	90	90 days	Large vessel, AC	GA: 69.21 (5.78) CS: 69.19 (6.46)	GA: 22(45.8) CS: 18(42.8)	GA: 9 (8–10) CS: 9 (8–10.25)	GA: 14 (11–16) CS: 14 (11–16)	GA: 21 (35.42) CS: 23 (47.62)	GA: 15(31.25) CS: 13(30.95)	GA: 17 (35.42) CS: 20 (47.62)
Schönenberger 2016 15	150	90 days	Large vessel, AC	GA: 71.8 (12.9) CS: 71.2 (14.7)	GA: 25 (34.2) CS: 35 (45.5)	GA: 8 (7–9) CS: 8 (6.25–9)	GA: 17 (13–20) CS: 17 (14–20)	GA: 40 (54.8) CS: 39 (50.6)	GA: 39 (53.4) CS: 43 (55.8)	GA: 53 (72.6) CS: 54 (70.1)
Simonsen 2018 13	128	90 days	Large vessel, AC	GA: 71.0 (10.0) CS: 71.8 (12.8)	GA: 29 (44.6) CS: 33 (52.4)	NA	GA: 18 (13–21) CS: 17 (15–21)	GA: 50 (76.9) CS: 51 (81.0)	GA: 21 (32.3) CS: 32 (50.8)	GA: 39 (60.0) CS: 32 (50.8)

Abbreviations: AC, Anterior Circulation; ASPECTS, Alberta Stroke Program Early Computed Tomography Score; CS, conscious sedation; GA, general anesthesia; M1, Middle cerebral artery M1 segment; mRS, modified Rankin scale; N, number of patients; NA, not available; NIHSS, National Institutes of Health Stroke Scale.

Notes: Scores on the NIHSS range from 0 to 42, with higher scores indicating a more severe deficit.

† Data presented as mean (standard deviation) or median (interquartile range).

### Pooled analysis of all studies

**Figure 2:**
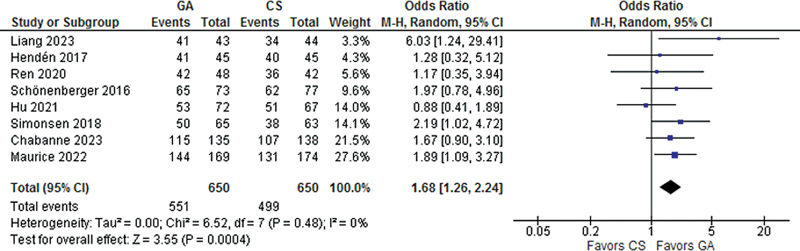
Recanalization success was significantly more common with general anesthesia compared with conscious sedation.

**Figure 3:**
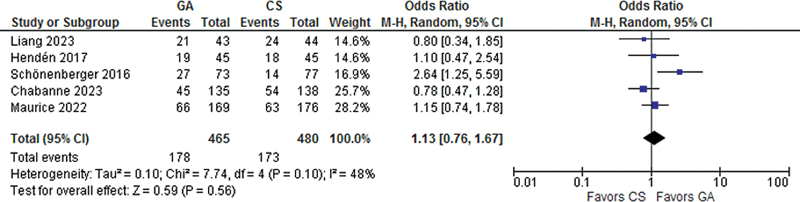
There was no significant difference between groups in good functional recovery.

**Figure 4:**
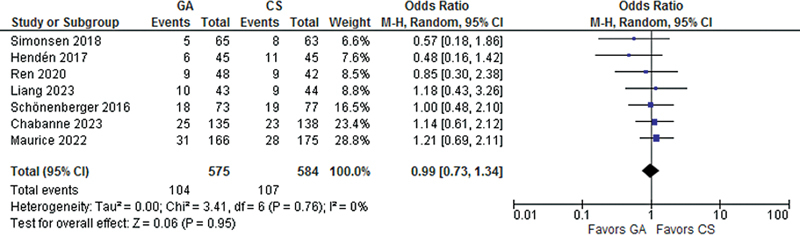
There was no significant difference between groups in Mortality at 3 months.

**Figure 5:**
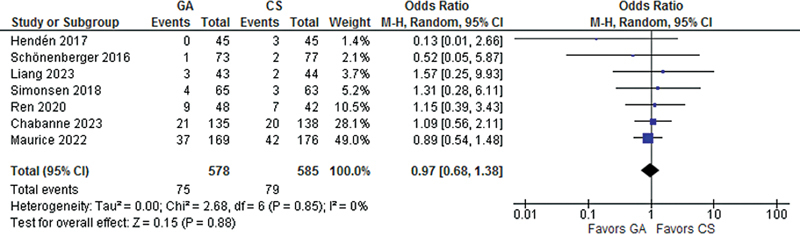
There was no significant difference between groups in cerebral hemorrhage.

Recanalization success was significantly higher in patients treated with GA as compared with CS (OR 1.68; 95% CI 1.26–2.24; *p* = 0.04; Figure 2 ). However, there was no significant difference between patients treated with GA and CS for good functional recovery (OR 1.13; 95% CI 0.76- 1.67; *p* = 0.56; Figure 3 ), three-month mortality (OR 0.99; 95% CI 0.73- 1.34; *p* = 0.95; Figure 4 ), and cerebral hemorrhage (OR 0.97; 95% CI 0.68- 1.38; *p* = 0.88); Figure 5 ). Similarly, the incidence of pneumonia was not statistically significant between groups (OR 1.23; 95% CI 0.56- 2.69; *p* = 0.61) ( Supplementary Material Figure S1 ).

### Sensitivity analysis and meta-regression

We performed a leave-one-out sensitivity analysis by systematically removing each study from the pooled estimate. After the removal of each study, the results for cerebral hemorrhage and three-month mortality were consistent. Recanalization success increased in the I ^2^ heterogeneity test after omitting Chabanne et al., 7 I ^2^ changing from 0% to 8%. Similarly, good functional recovery was sensitive to the removal of Hendén et al. 14 and Maurice et al., 3 with a change in I ^2^ heterogeneity from 48% to 61% and 61%, respectively. By omitting Schönenberger, 15 the heterogeneity in the endpoint was eliminated (OR 0.96; 95% CI 0.72–1.28; I2 = 0%). A comprehensive result of all sensitivity analyses conducted in the primary endpoints is shown in Supplementary Material Tables S2–S5 .

Prespecified Meta-regression showed no significant interaction between recanalization success and the mean NIHSS score. We could only analyze seven studies due to the availability of NIHSS scores data; hence, this adaptation is restricted and impotent. The results of this analysis are available in the Supplementary Material Figure S2 .

### Quality and evidence assessment

Most included studies were judged to be at low risk of bias except for three studies, 3 13 16 which were judged as having “some concerns” in 3 separate domains: randomization process, deviations from the intended interventions, and selection of reported results ( Supplementary Material Figure S3 ).

On funnel plot analyses, the studies demonstrated a symmetrical distribution by their weight and conversion toward the pooled effect as the weight increased; thus, there was no definitive evidence of publication bias in the funnel plots. ( Supplementary Material Figure S4 ). The overall GRADE assessment certainty was high ( Supplementary Material Figure S5 ).

## DISCUSSION

In this systematic review and meta-analysis of 8 studies and 1300 patients, we compared GA with CS in the mechanical thrombectomy for AIS due to LVO. We found that recanalization rates were higher in patients with GA (OR 1.68; 95% CI 1.26–2.24; *p* < 0.04), but there was no difference between groups regarding functional recovery, three-month mortality, cerebral hemorrhage, and pneumonia.

A recent meta-analysis 6 also reported significantly higher recanalization rates in patients under GA; however, this meta-analysis included only preliminary data from the trial by Liang et al. 11 and did not include a recently published RCT. Our meta-analysis aligns with previous prospective studies that showed worse results in patients treated with CS. It is hypothesized that GA's significant hazards rely on inadvertent hypotension during induction and maintenance of GA; impaired cerebral autoregulation blood flow from cerebral ischemia potentiated by these anesthetic drug effects may also play a role, 17 which could explain our results.

Although the GA group had a higher recanalization rate, there was no statistically significant difference in functional recovery. Considering there was no difference in pneumonia rates, this may be related to other factors, like NIHSS, age, and time to reperfusion. The meta-regression analysis showed no interaction between the NIHSS score and recanalization rate. There wasn't enough data on patients' ages, so it was not possible to evaluate it in this meta-analysis.

To our knowledge, this is the first meta-analysis to evaluate pneumonia occurrence associated with mechanical thrombectomy. Prospective studies 18 have shown a higher rate of pneumonia in patients with LVO during acute care; moreover, the risk could further increase in cases where mechanical thrombectomy is performed, and patients undergo intensive care unit (ICU) treatment. Pneumonia could result in higher mortality, as well as a higher length of stay and hospitalization costs. However, a prospective study 19 did not find statistically significant higher pneumonia rates in patients intubated for mechanical thrombectomy. The 4 RCTs that disclosed pneumonia rates between GA and CS had indistinguishable statistical differences between either anesthetic choice. This could also be related to the absence of difference in functional recovery found in our study.

Only one trial included in this meta-analysis assessed both anterior and posterior circulation strokes, so it was not possible to evaluate for differences between GA and CS in these groups.

Our study has some limitations. First, there were slight differences between groups concerning the study population; however, there was no significant heterogeneity in the analyses. We included a sensitivity analysis which showed similar results for cerebral hemorrhage, three-month mortality, and recanalization success; there was moderate heterogeneity in good functional recovery, which could be explained because one study 3 evaluated the functional status in a more extended period (from 2 to 6 months of the stroke). Funnel plots did not encounter signs of publication bias in this meta-analysis. Second, our study does not apply to all mechanical thrombectomy patients since most studies included only anterior circulation strokes, and only one study of this meta-analysis included posterior circulation strokes. Third, protocols for GA and CS differed between studies.

GA provides higher recanalization rates than CS. However, no difference is significant regarding good functional outcome, three-month mortality, cerebral hemorrhage, and pneumonia, suggesting that either approach can safely be chosen based on other patient characteristics.
