# Supplementary material for: General anesthesia versus conscious sedation in mechanical thrombectomy for patients with acute ischemic stroke: systematic review and meta-analysis
Source: Arq Neuropsiquiatr. 2024 Apr 12;82(4):s00441785693. doi: 10.1055/s-0044-1785693 (PMC11014755; doi:10.1055/s-0044-1785693)
Supplement: Supplementary file 1 — Supplementary Material [file 10-1055-s-0044-1785693-s230290.pdf]

Supplementary Material

Supplementary Table S1 Search strategy

Cochrane	((“Acute Ischemic Stroke”):ti,ab,kw OR (“ischemic stroke”):ti,ab,kw OR (“posterior circulation stroke”):ti,ab,kw OR (“endovascular therapy”):ti,ab,kw OR (“endovascular treatment”):ti,ab,kw OR (thrombectomy):ti,ab,kw OR (“vertebrobasilar stroke”):ti,ab,kw) AND (“general anesthesia” OR “General Anesthesias”) AND (“Non- general anesthesia” OR “conscious sedation” OR “Moderate Sedation” OR “local anesthesia” OR “anesthesia care”)
Embase	(TITLE-ABS-KEY(“Acute Ischemic Stroke”) OR TITLE-ABS-KEY(“ischemic stroke”) OR TITLE-ABS-KEY(“posterior circulation stroke”) OR TITLE-ABS-KEY(“endovascular therapy”) OR TITLE-ABS-KEY(“endovascular treatment”) OR TITLE-ABS-KEY (thrombectomy) OR TITLE-ABS-KEY(“vertebrobasilar stroke”)) AND (“general anesthesia” OR “General Anesthesias”) AND (“Non-general anesthesia” OR “conscious sedation” OR “Moderate Sedation” OR “local anesthesia” OR “anesthesia care”)
PubMed	(“Acute Ischemic Stroke” OR “ischemic stroke” OR “posterior circulation stroke” OR “endovascular therapy” OR “endovascular treatment” OR thrombectomy OR “vertebrobasilar stroke”) AND (“general anesthesia” OR “General Anesthesias”) AND (“Non- general anesthesia” OR “conscious sedation” OR “Moderate Sedation” OR “local anesthesia” OR “anesthesia care”)

Supplementary Table S2 Sensitivity analysis for recanalization success

Study	OR	95%-CI	I^2
Omitting Chabanne	1.69	(1.19–2.28)	8%
Omitting Hendén	1.70	(1.25–2.32)	6%
Omitting Hu	1.87	(1.37–2.55)	0%
Omitting Liang	1.61	(1.20–2.16)	0%
Omitting Maurice	1.61	(1.14–2.29)	5%
Omitting Ren	1.72	(1.27–2.33)	2%
Omitting Schonenberger	1.65	(1.20–2.27)	6%
Omitting Simonsen	1.61	(1.18–2.20)	0%
**Including all studies**	**1.68**	**(1.26–2.24)**	**0%**

Supplementary Table S3 Sensitivity analysis for good functional recovery

Study	OR	95%-CI	I^2
Omitting Chabanne	1.28	(0.81–2.01)	42%
Omitting Hendén	1.14	(0.70–1.85)	61%
Omitting Liang	1.20	(0.76–1.91)	58%
Omitting Maurice	1.13	(0.64–2.01)	61%
Omitting Schonenberger	0.96	(0.72–1.28)	0%
**Including all studies**	**1.13**	**(0.76–1.67)**	**48%**

Supplementary Table S4 Sensitivity analysis for 3-month mortality

Study	OR	95%-CI	I^2
Omitting Chabanne	0.95	(0.67–1.34)	0%
Omitting Hendén	1.05	(0.77–1.44)	0%
Omitting Liang	0.97	(0.71–1.34)	0%
Omitting Maurice	0.92	(0.64–1.31)	0%
Omitting Ren	1.01	(0.73–1.38)	0%
Omitting Schonenberger	0.99	(0.71–1.38)	0%
Omitting Simonsen	1.03	(0.75–1.41)	0%
**Including all studies**	**0.99**	**(0.73–1.34)**	**0%**

 © 2024. The Author(s).

Supplementary Table S5 Sensitivity analysis for cerebral hemorrhage

Study	OR	95%-CI	I^2
Omitting Chabanne	0.93	(0.62–1.41)	0%
Omitting Hendén	1.00	(0.70–1.43)	0%
Omitting Liang	0.96	(0.67–1.37)	0%
Omitting Maurice	1.06	(0.64–1.73)	0%
Omitting Ren	0.95	(0.66–1.38)	0%
Omitting Schonenberger	0.99	(0.69–1.41)	0%
Omitting Simonsen	0.96	(0.67–1.37)	0%
**Including all studies**	0.97	(0.68–1.38)	0%

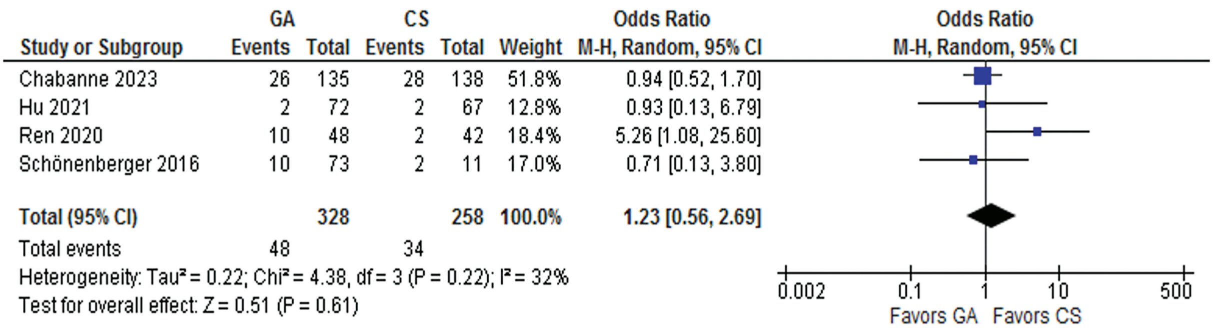

Supplementary Fig. S1 There was no significant difference between groups in pneumonia. Abbreviations: GA, general anesthesia; CS, conscious sedation.

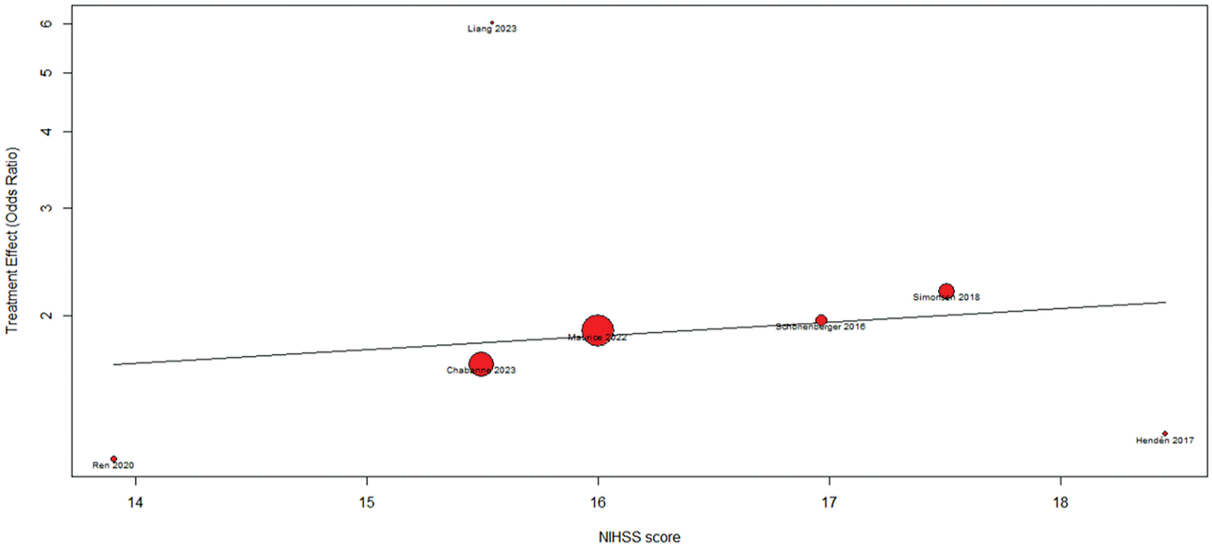

Supplementary Fig. S2 Meta Regression assessing the impact of NIHSS score proportion on recanalization success.

	Effect estimate	p-value	I^2	Test for residual heterogeneity
Intercept	−0.2029	0.9341	0.00%	$p = 0.6757$
NIHSS score	0.0513	0.7345		

Abbreviation: NIHSS, National Institutes of Health Stroke Scale.

 © 2024. The Author(s).

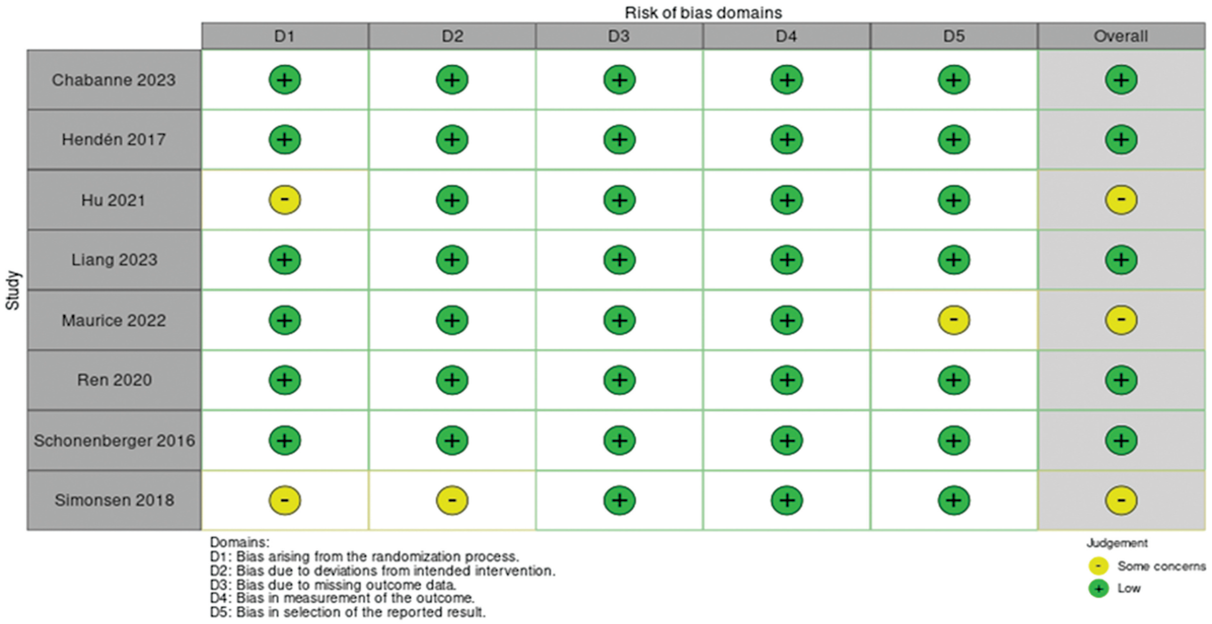

Supplementary Fig. S3 Critical appraisal of individual studies according to the Cochrane Collaboration's tool for assessing risk of bias in randomized trials.

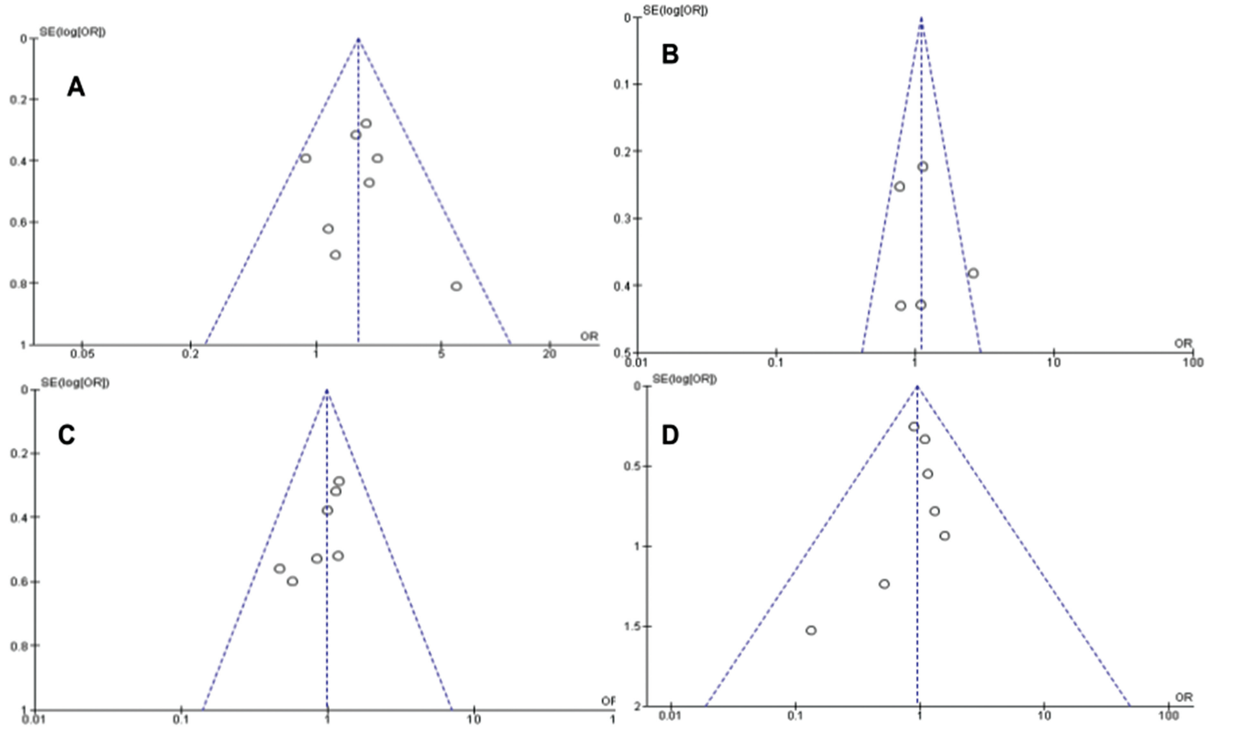

Supplementary Fig. S4 Funnel plot for recanalization success (A), good functional recovery (B), 3- month mortality (C), and cerebral hemorrhage (D) showed no definitive evidence of publication bias.

 © 2024. The Author(s).

Author(s):
Question: General Anesthesia compared to Conscious sedation for Acute Ischemic Stroke
Setting:
Bibliography:

Certainty assessment							№ of patients		Effect		Certainty	Importance
**№ of studies**	**Study design**	**Risk of bias**	**Inconsistency**	**Indirectness**	**Imprecision**	**Other considerations**	**General Anesthesia**	**Conscious sedation**	**Relative (95% CI)**	**Absolute (95% CI)**		
**Recanalization Success**												
8	randomised trials	not serious	not serious	not serious	not serious	none	551/650 (84.8%)	499/650 (76.8%)	**OR 1.68** (1.26 to 2.24)	**80 more per 1.000** (from 39 more to 113 more)	⊕⊕⊕⊕ High	
**Good functional recovery**												
5	randomised trials	not serious	serious[a]	not serious	not serious	none	178/465 (38.3%)	173/480 (36.0%)	**OR 1.13** (0.76 to 1.67)	**29 more per 1.000** (from 61 fewer to 124 more)	⊕⊕⊕◯ Moderate	
**3-Month Mortality**												
7	randomised trials	not serious	not serious	not serious	not serious	none	104/575 (18.1%)	107/584 (18.3%)	**OR 0.99** (0.73 to 1.34)	**1 fewer per 1.000** (from 43 fewer to 48 more)	⊕⊕⊕⊕ High	
**Cerebral hemorrhage**												
7	randomised trials	not serious	not serious	not serious	not serious	none	75/578 (13.0%)	79/585 (13.5%)	**OR 0.97** (0.68 to 1.38)	**4 fewer per 1.000** (from 39 fewer to 42 more)	⊕⊕⊕⊕ High	

CI: confidence interval; **OR:** odds ratio

Explanations

a. Moderate heterogeneity(I2 = 48%). Downgraded by one level for inconsistency.

Supplementary Fig. S5 GRADE assessment.

 © 2024. The Author(s).
